# Unveiling the Systemic Impact of Congestion in Heart Failure: A Narrative Review of Multisystem Pathophysiology and Clinical Implications

**DOI:** 10.3390/jcdd12040124

**Published:** 2025-03-31

**Authors:** Daniela Mocan, Radu Jipa, Daniel Alexandru Jipa, Radu Ioan Lala, Florin Claudiu Rasinar, Iulia Groza, Ronela Sabau, Damaris Sulea Bratu, Diana Federica Balta, Sergiu Teodor Cioban, Maria Puschita

**Affiliations:** 1 Multidisciplinary Doctoral School, Vasile Goldis Western University of Arad, 310025 Arad, Romania; mocandaniela3@gmail.com (D.M.); radu_lala@yahoo.com (R.I.L.); mpuschita.mp@gmail.com (M.P.); 2 Research Center of the Institute of Cardiovascular Diseases Timisoara, 300310 Timisoara, Romania; 3 Department VII, Internal Medicine II, Discipline of Cardiology, University of Medicine and Pharmacy “Victor Babes” Timisoara, E. Murgu Square, Nr. 2, 300041 Timisoara, Romania; 4 Institute of Cardiovascular Diseases of Timisoara, 300310 Timisoara, Romania; 5 Faculty of Medicine, Department of “Life Sciences”, Vasile Goldis Western University of Arad, Romania 86, Liviu Rebreanu Street, 310048 Arad, Romania; 6 Arad County Clinical Emergency Hospital, 310037 Arad, Romania; 7 Doctoral School, Victor Babes University of Timisoara, 300041 Timisoara, Romania; daniel.jipa@umft.ro; 8 Victor Babes Clinical Hospital for Infectious Diseases and Pneumology of Timisoara, 300041 Timisoara, Romania

**Keywords:** heart failure, congestion, pathophysiology

## Abstract

Congestion is a key clinical feature of heart failure (HF), contributing to hospitalizations, disease progression, and poor outcomes. While traditionally considered a hemodynamic issue, congestion is now recognized as a systemic process affecting multiple organs. Renal dysfunction arises from impaired perfusion and sodium retention, leading to maladaptive left ventricular remodeling. Hepatic congestion contributes to cholestatic liver injury, while metabolic disturbances drive anemia, muscle wasting, and systemic inflammation. Additionally, congestion disrupts the intestinal barrier and immune function, exacerbating HF progression. Given its widespread impact, effective congestion management requires a shift from a cardiovascular-centered approach to a comprehensive, multidisciplinary strategy. Targeted decongestive therapy, metabolic and nutritional optimization, and immune modulation are crucial in mitigating congestion-related organ dysfunction. Early recognition and intervention are essential to slow disease progression, preserve functional capacity, and improve survival. Addressing HF congestion through personalized, evidence-based strategies is vital for optimizing long-term care and advancing treatment paradigms.

## 1. Introduction

Heart failure (HF) is a complex syndrome in which congestion plays a central role in disease progression and clinical outcomes. Characterized by extracellular fluid accumulation, congestion is the leading cause of hospitalizations and readmissions, affecting patients across various HF phenotypes, from de novo HF to acute decompensation and advanced HF [1,2,3].

The etiology of HF is diverse, with ischemic heart disease as the most common cause, often linked to prior myocardial infarction [4,5]. Non-ischemic factors such as hypertension, diabetes, obesity, chronic lung disease, systemic inflammation, and cardiotoxic agents also contribute, frequently interacting with arrhythmias, infections, and comorbidities, leading to varied clinical presentations [6,7]. Clinically, HF presents in various forms, from acute pulmonary edema and hypertensive HF to severe cases like cardiogenic shock and decompensated chronic HF. Prognosis varies, with hypertension-related HF showing better outcomes, while cardiogenic shock remains the most fatal presentation [6,8,9].

While HF originates in the heart, persistent congestion and impaired perfusion impact multiple organ systems, including the kidneys, liver, lungs, gastrointestinal tract, and nervous system. This systemic involvement, driven by congestion, inflammation, and neurohormonal activation, worsens morbidity and mortality, complicating management [8]. Recognizing the multi-organ impact of HF-related congestion is essential for optimizing treatment and improving patient outcomes.

## 2. Congestion Is a Distinctive Mark of Heart Failure

### 2.1. Clinical Profile of Patients and Symptomatology

Heart failure (HF) classification has evolved, yet defining distinct patient groups remains challenging. Table 1 presents the most commonly used classification systems. One of the most accepted classifications is the Universal Classification, which categorizes HF into four progressive stages. Stage A includes individuals at risk but without structural heart disease, while Stage B, or pre-HF, involves structural abnormalities without symptoms. Stage C marks the onset of symptomatic HF, and Stage D represents advanced disease with persistent symptoms despite optimal treatment [6,10]. Ejection fraction remains central to HF classification, distinguishing between preserved (HFpEF), mildly reduced (HFmrEF), and reduced ejection fraction (HFrEF). The NYHA functional classification further assesses severity based on physical limitations, ranging from asymptomatic (Class I) to severe symptoms even at rest (Class IV) [11].

The clinical presentation of HF varies depending on which side of the heart is affected. Left-sided HF leads to pulmonary congestion, causing breathlessness, fatigue, and cough. In contrast, right-sided HF results in systemic congestion, often manifesting as peripheral edema, hepatomegaly, and jugular vein distension. While right HF can develop as a consequence of left HF, it may also arise from pulmonary diseases or congenital heart defects, further complicating disease progression [12,13,14].

### 2.2. Pathophysiology of Congestion in HF

**Figure 1:**
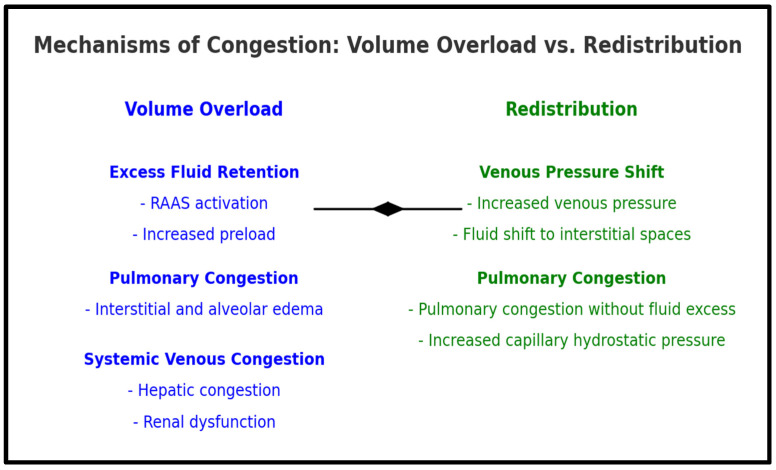
**Mechanisms of congestion: volume overload vs. redistribution.** This figure illustrates the two main mechanisms of congestion in acute heart failure. Volume overload results from excess fluid retention (RAAS activation, increased preload), leading to pulmonary (interstitial/alveolar edema) and systemic venous congestion (hepatic congestion, renal dysfunction). Redistribution occurs via a venous pressure shift, increasing capillary hydrostatic pressure and causing pulmonary congestion without fluid excess.

Congestion is a primary contributor to acute heart failure (AHF) hospitalizations, often outweighing symptoms related to reduced cardiac output [15]. It develops through two distinct mechanisms: volume overload and redistribution, each with unique pathophysiological implications as summarized in Figure 1.

Volume overload occurs gradually over days or weeks as reduced cardiac output activates neurohormonal compensatory mechanisms, including renin-angiotensin-aldosterone system (RAAS) and sympathetic nervous system (SNS) activation, leading to vasoconstriction, sodium and water retention, and increased preload and afterload [16,17,18,19]. The neuroendocrine system triggers the release of epinephrine, norepinephrine, endothelin-1 (ET-1), and vasopressin, all of which induce vasoconstriction [16]. This increase in vascular resistance raises afterload, exacerbating the heart’s workload [17]. In addition, elevated levels of cyclic adenosine monophosphate (cAMP) and cytosolic calcium in myocytes can impair myocardial relaxation, leading to modifications in myocyte regeneration, myocardial hypertrophy, and hypercontractility [18]. The paradoxical requirement for heightened cardiac output to fulfill myocardial demands ultimately results in myocardial cell death and apoptosis. Once these compensatory mechanisms become exhausted, maladaptation ensues. Reduced renal perfusion further activates the sympathetic nervous system, leading to RAAS stimulation. This cascade results in systemic vasoconstriction and sodium and water retention, thereby increasing both preload and afterload. The release of antidiuretic hormone (ADH) further exacerbates fluid retention. Additionally, RAAS activation leads to the production of angiotensin II, a potent vasoconstrictor that has been shown to stimulate myocardial hypertrophy and interstitial fibrosis, contributing to myocardial remodeling [13,17,18,19].

The fluid accumulation that leads to decompensated heart failure begins within the intravascular compartment. Sustained high hydrostatic pressures in capillaries result in tissue congestion, progressing through a sequence of identifiable stages. Initially, congestion is hemodynamic, characterized by rising venous pressures. As fluid accumulates in the lungs and liver, it progresses to clinical congestion, presenting with overt symptoms [19].

Volume redistribution, in contrast, is a rapid shift of fluid from the splanchnic venous system to the central circulation, often triggered by hypertension, myocardial ischemia, renal dysfunction, or neurohormonal activation [16,17]. This process elevates central venous and pulmonary pressures, leading to acute pulmonary congestion without significant weight gain [17,20,21,22]. In such cases, vasodilator therapy is often more effective than diuretics in symptom management [17].

Recognizing these mechanisms is essential for optimizing therapeutic strategies, as targeted interventions can improve clinical outcomes in patients with HF.

## 3. Congestion and Organ Response

**Figure 2:**
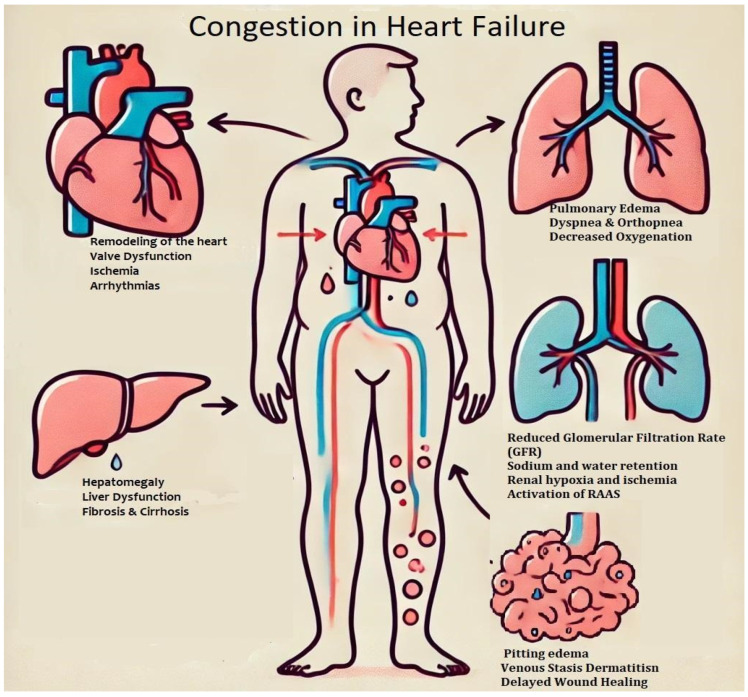
**Congestion in heart failure.** Congestion in heart failure results from increased venous pressure and fluid retention, leading to multisystem involvement. Pulmonary congestion manifests as pulmonary edema, dyspnea, and impaired oxygenation. Renal dysfunction occurs due to reduced glomerular filtration rate (GFR), sodium and water retention, and activation of the renin-angiotensin-aldosterone system (RAAS). Hepatic congestion can lead to hepatomegaly, liver dysfunction, and fibrosis. Peripheral congestion contributes to pitting edema and venous stasis dermatitis. Meanwhile, structural and functional cardiac changes, including remodeling, valve dysfunction, and ischemia, further exacerbate heart failure progression.

Congestive heart failure is considered a systemic condition, affecting not only the heart but also contributing to renal dysfunction due to reduced perfusion pressure, sodium retention, and subsequent subendocardial ischemia, which leads to left ventricular remodeling [23]. Additionally, it can cause cholestatic liver injury, anemia, muscle damage due to metabolic disturbances, and various other complications [24]. Figure 2 depicts venous congestion in heart failure and its systemic consequences.

### 3.1. Effects of Congestion on the Heart

#### 3.1.1. Congestion and Heart Remodeling

As venous congestion increases in heart failure (HF), the heart faces higher preload, leading to greater ventricular wall stress. Initially, the heart compensates by stretching its chambers and thickening its walls to maintain output [13,15,25,26]. Natriuretic peptides are released to help manage the overload, but this relief is temporary. Over time, what starts as an adaptation turns into a pathway toward heart failure [19].

The left ventricle undergoes significant changes, enlarging and becoming more spherical, a sign of worsening HF. These changes are part of various patterns of left ventricular (LV) remodeling, including concentric and eccentric hypertrophy, which often precede dysfunction and dilation. As remodeling progresses, myocytes hypertrophy, but persistent congestion leads to myocardial apoptosis, fibrosis, and loss of contractility, worsening LV dysfunction [19,27].

In a four-year study by Wolfgang Lieb, similar remodeling patterns were observed in patients with obesity and hypertension [28]. Ciampi and Villari classified heart failure into several phenotypes, including the following:

- The Weak Heart: Systolic dysfunction with LV dilation and reduced ejection fraction (HFrEF).
- The Big Heart: Remodeling from hypertension or cardiomyopathies with LV dilation and mitral regurgitation.
- The Noisy Heart: Mitral regurgitation due to LV distortion in dilated or ischemic cardiomyopathy.
- The Stiff Heart: Diastolic dysfunction with elevated filling pressures.
- The Wet Heart: Pulmonary congestion and edema, visible on imaging [29].

#### 3.1.2. Valves Disfunction

As the heart chambers enlarge, the mitral and tricuspid valves struggle to function properly. Geometric distortion from remodeling prevents proper valve coaptation, causing functional mitral and tricuspid regurgitation. This allows blood to flow backward, worsening congestion and creating a cycle of volume overload [15]. What started as minor distortion leads to significant valvular insufficiency, making it harder for the heart to generate forward flow and exacerbating pulmonary and systemic congestion [30].

#### 3.1.3. Ischemia and Arrhythmias

A congested heart is oxygen-starved, as rising ventricular pressures compress the coronary microcirculation, limiting oxygen delivery to the myocardium. This causes subendocardial ischemia, leading to fibrosis, scarring, and impaired contractility [19]. Congestion also disrupts electrical stability, with ischemia and fibrosis increasing the risk of arrhythmias, especially atrial fibrillation (AF). The dilated left atrium increases AF risk, reducing atrial contraction and worsening congestion. Elevated ventricular rates worsen left ventricular function, creating a cycle of heart failure and arrhythmia. Arrhythmias are common in HF, with 20–80% of patients experiencing non-sustained ventricular tachycardia (VT), which can lead to palpitations, syncope, or sudden cardiac death [19].

#### 3.1.4. The Right Ventricle Dysfunction

The right ventricle (RV), historically considered a “passive chamber” and often overshadowed by the left ventricle (LV), plays a crucial role in the progression of heart failure (HF) [31]. While the LV has traditionally been the primary focus in HF research and management, RV dysfunction is a key determinant of prognosis [32,33,34].

In many cases, RV failure develops secondary to increased pulmonary pressures resulting from LV dysfunction, eventually leading to biventricular HF, a condition associated with significantly worse outcomes [31]. As pulmonary congestion increases pulmonary artery pressures, the thin-walled RV faces an increasing afterload burden, making it more vulnerable to decompensation [15].

The RV differs from the LV in embryological origin, geometry, myocardial composition, and hemodynamic function, operating at lower pressures and being more volume-dependent [31]. These distinctions suggest that the RV responds uniquely to hemodynamic stress, making its failure pathophysiologically distinct from that of the LV. One of the most common causes of RV dysfunction is pulmonary hypertension (PH), a condition characterized by elevated pulmonary vascular resistance (PVR) [15,35]. In the early stages, the RV compensates through hypertrophy, but as pressure overload persists, maladaptive remodeling occurs, leading to RV dilation, contractile dysfunction, and eventual failure [15,32,34,36].

Pulmonary hypertension arises from various underlying conditions. In patients with chronic lung diseases such as chronic obstructive pulmonary disease (COPD) and interstitial lung disease, hypoxia-induced vasoconstriction and pulmonary vascular remodeling elevate pulmonary pressures, placing excessive stress on the RV [15,32]. Other significant contributors to RV dysfunction include chronic thromboembolic disease, which leads to persistent vascular obstruction, as well as right ventricular myocardial infarction (RVMI), resulting in ischemic injury and impaired contractility. Additionally, arrhythmogenic right ventricular cardiomyopathy (ARVC), a genetic disorder characterized by progressive fibrofatty myocardial replacement, increases the risk of RV failure and life-threatening arrhythmias [15,32,37].

As RV dysfunction progresses to right-sided heart failure, systemic venous congestion ensues, leading to hepatic congestion, ascites, and worsening peripheral edema. These systemic consequences extend the impact of RV failure beyond the heart, contributing to multiorgan dysfunction and poor clinical outcomes [19,38]. Given its significant role in HF progression, early recognition and targeted interventions for RV dysfunction are essential in improving patient outcomes [34].

#### 3.1.5. The Need for Early Intervention and Treatment

Congestion fuels the progression of heart failure, accelerating cardiac remodeling, dilation, and ischemia. Recognizing it early is crucial to breaking the cycle. Echocardiographic markers and advanced techniques like speckle tracking can identify early dysfunction before symptoms worsen [13,39]. However, imaging alone is not enough. Proactive treatment with decongestive therapy, volume control, and neurohormonal blockade is essential to preserve heart function and prevent further damage [15].

### 3.2. Effects of Congestion in the Lungs

Pulmonary congestion in heart failure (HF) results from elevated left atrial pressure, leading to fluid leakage into the interstitial and alveolar spaces. This disrupts gas exchange, causing dyspnea and respiratory symptoms [13,15,40].

Increased left ventricular filling pressures heighten pulmonary capillary permeability, exacerbated by mitral regurgitation, leading to alveolar edema and pleural effusion when lymphatic drainage is overwhelmed [15,41]. Persistent congestion disrupts Starling forces, contributing to cardiopulmonary remodeling, endothelial dysfunction, and fibrosis, impairing alveolar diffusion and promoting pulmonary vasoconstriction [42,43]. Over time, these changes result in pulmonary hypertension and restrictive lung dysfunction, further worsening respiratory capacity and HF progression.

Beyond fluid accumulation and impaired gas exchange, pulmonary congestion in heart failure (HF) leads to further complications that accelerate disease progression.

- Respiratory Complication: Pulmonary congestion reduces lung compliance, increasing breathing effort and causing hypoxemia and respiratory distress [44]. Impaired mucociliary clearance heightens pneumonia risk, while ventilation-perfusion mismatch worsens oxygen exchange, triggering compensatory mechanisms such as increased respiratory rate and sympathetic activation [26,45,46]. Many HF patients also experience sleep-disordered breathing, including Cheyne–Stokes respiration and central sleep apnea, contributing to nocturnal hypoxia, fatigue, and cognitive decline [44].
- Right Heart Strain and Pulmonary Hypertension: Persistent pulmonary congestion raises pulmonary vascular resistance, increasing the right ventricular workload and leading to hypertrophy and eventual right-sided heart failure. In severe cases, pulmonary hypertension develops, further impairing gas exchange and reducing exercise tolerance [42,43,47].

#### Diagnostic and Therapeutic Approaches

Pulmonary congestion is identified through imaging techniques such as chest X-rays (detecting pulmonary edema and Kerley B lines), lung ultrasound (revealing B-lines), and echocardiography (assessing left atrial and pulmonary artery pressures) [15,47]. Persistent congestion increases morbidity and mortality, necessitating early intervention.

Management focuses on reducing pulmonary venous pressure and fluid overload using diuretics, vasodilators, and non-invasive ventilation (CPAP/BiPAP) in acute cases. Preventive strategies include optimizing HF management and monitoring for respiratory infections to prevent complications [42,44,48].

### 3.3. Interdependence Between Renal Function and Heart Failure

#### 3.3.1. Kidney and Congestion

The interplay between the heart and kidneys is fundamental to fluid balance and circulatory homeostasis. Any dysfunction in one organ inevitably influences the other, forming a pathophysiological cycle that exacerbates cardiorenal syndrome. Heart failure frequently coexists with chronic kidney disease (CKD), with nearly 49% of HF patients exhibiting some degree of renal impairment [49,50]. Persistent congestion in this setting contributes to diuretic resistance, electrolyte imbalances, hypotension, and systemic inflammation, all of which worsen outcomes [51,52,53].

Renal congestion in heart failure is a multifaceted problem. Primary kidney disease, such as glomerulonephritis or diabetic nephropathy, reduces the glomerular filtration rate (GFR), impairing the excretion of sodium and water. In response, the RAAS is activated, leading to sodium retention, volume overload, and hypertension. Proteinuria, common in CKD, further lowers oncotic pressure, allowing fluid to shift into the interstitial space, worsening edema and congestion [23]. On the other hand, chronic heart failure (CHF) leads to progressive renal dysfunction due to declining cardiac output and rising central venous pressure (CVP). Increased renal venous congestion diminishes glomerular perfusion, disrupting the pressure gradient between afferent and efferent arterioles. This results in impaired sodium handling, water retention, and worsening congestion, setting off a vicious cycle of progressive kidney and heart dysfunction [54,55].

The decline in GFR is a well-established marker of poor prognosis in HF [56,57]. Left ventricular dysfunction impairs renal perfusion, activating RAAS and the SNS, further vasoconstricting afferent arterioles, and reducing GFR [58,59,60]. Right ventricular failure, in contrast, leads to elevated CVP, increasing renal interstitial pressure and causing tubular compression, which exacerbates sodium and water retention, ultimately worsening systemic congestion [56].

Chronic renal congestion in heart failure (HF) contributes to acute kidney injury (AKI) due to medullary hypoxia and tubular necrosis, often worsened by aggressive diuresis or hypotension [26]. Persistent venous hypertension promotes inflammation, fibrosis, and oxidative stress, leading to renal dysfunction [57,61]. Impaired clearance increases drug toxicity risks, while electrolyte imbalances and systemic inflammation accelerate endothelial dysfunction and myocardial remodeling [62]. Reduced erythropoietin (EPO) production leads to anemia, exacerbating fatigue, hypoxia, and cardiac strain, further worsening HF outcomes [24,61]. All these effects of chronic congestion are summarized in Table 2.

#### 3.3.2. Assessing and Managing Renal Congestion in Heart Failure

Renal congestion assessment requires hemodynamic and imaging techniques. While CVP monitoring is useful, it may not accurately reflect intrarenal congestion. Intrarenal Doppler ultrasonography, particularly intrarenal venous flow (IRVF) analysis, correlates with right atrial pressure and predicts prognosis. Abnormal IRVF patterns are linked to higher 1-year mortality in HF patients. Contrast-enhanced ultrasonography (CEUS) offers direct visualization of renal microvascular perfusion, with studies showing improved renal outcomes following decongestive therapy [63,64].

### 3.4. Effects of Congestion on the Digestive System

#### 3.4.1. Liver and Congestion

One of the most prominent consequences of congestion is hepatic involvement, commonly referred to as congestive hepatopathy. The liver becomes engorged due to elevated central venous pressure, which is transmitted to the sinusoidal capillaries. Because the hepatic venous system lacks valves, venous pressure is directly transmitted, causing sinusoidal congestion, peri-sinusoidal edema, and hepatocyte hypoxia. This process reduces oxygen diffusion to hepatocytes, impairing liver metabolism and detoxification functions [65,66,67]. Laboratory markers often show elevated alkaline phosphatase, bilirubin, and γ-glutamyltransferase (GGT) due to cholestasis and hepatocyte stress. Over time, chronic congestion may result in fibrosis and progression to cardiac cirrhosis. Although liver dysfunction in HF is often asymptomatic, routine biochemical testing frequently reveals elevations in right atrial pressure, severe tricuspid regurgitation, and serum natriuretic peptides, all of which correlate with worsening hepatic congestion [66,67].

Chronic passive congestion may also impair hepatic synthetic function, leading to prolonged prothrombin time and hypoalbuminemia, further contributing to edema formation [68].

#### 3.4.2. Intestinal and Congestion

In heart failure (HF), intestinal function is significantly impacted by splanchnic venous congestion, low cardiac output, and ischemia, leading to symptoms like nausea, bloating, and anorexia, often worsened by HF medications [69,70]. Impaired circulation causes malabsorption, exacerbating malnutrition and weight loss. Additionally, changes in gut microbiota play a critical role in HF progression. Weakened intestinal barriers allow bacteria and endotoxins to enter the bloodstream, triggering systemic inflammation and worsening cardiac and renal function [59,71]. This “leaky gut” effect, along with microbial dysbiosis, contributes to increased inflammation and metabolic dysfunction, accelerating HF and cachexia development [66,71]. The malabsorption of key nutrients worsens weight loss and instability, and cardiac cachexia, marked by muscle wasting and metabolic decline, remains a strong predictor of poor outcomes in HF [65,72].

These interconnected issues highlight the need for targeted interventions to manage both gastrointestinal and systemic complications in HF patients.

#### 3.4.3. Assessment and Management of Digestive Congestion in HF

Gastrointestinal involvement in heart failure (HF) is often overlooked. While liver dysfunction can be assessed through hepatic enzyme tests and imaging, intestinal impairment remains challenging to evaluate [65,73,74].

Treatment focuses on relieving congestion while preserving gut and liver function. Diuretics aid in managing hepatic and splanchnic congestion, though excessive use may worsen ischemia and malabsorption [73]. Nutritional strategies, including micronutrient supplementation and gut microbiome modulation, are essential for preventing cachexia. Probiotics and prebiotics are emerging as potential therapies to restore microbial balance and reduce inflammation [74].

### 3.5. Effects of Congestion in Heart Failure on the Neurovascular System

Congestion in heart failure (HF) significantly impacts the neurovascular system, affecting both central and peripheral circulation. The interplay of elevated venous pressure, endothelial dysfunction, cerebral hypoperfusion, and autonomic dysregulation contributes to neurological decline and vascular complications, worsening disease severity [75,76].

Reduced cerebral perfusion due to impaired cardiac output disrupts blood flow regulation, increasing the risk of cognitive impairment, white matter lesions, and stroke. Chronic hypoxia and neuroinflammation further accelerate neurodegeneration, leading to deficits in memory, executive function, and processing speed, resembling vascular dementia [77]. Advanced HF and atrial fibrillation further elevate stroke risk [76,77].

HF-associated autonomic imbalance enhances sympathetic activity while weakening parasympathetic control, resulting in baroreflex impairment, vascular resistance elevation, and unstable blood pressure regulation. Increased angiotensin II and norepinephrine levels exacerbate vasoconstriction, hypertension, and endothelial dysfunction, contributing to arrhythmias and sudden cardiac death [16,78,79].

Congestion also affects peripheral circulation, leading to endothelial dysfunction, vascular stiffness, and reduced blood flow, causing symptoms like claudication, fatigue, and neuropathy [80]. Retinal microvascular changes, including venous dilation and hypertensive retinopathy, serve as markers of systemic vascular damage and HF severity [77,81].

Managing neurovascular dysfunction in HF requires optimizing cardiac output, reducing congestion, and correcting neurohormonal imbalances. Beta-blockers, ACE inhibitors, and aldosterone antagonists help regulate sympathetic overactivity and improve vascular function. Routine cognitive assessments, neuroimaging, and autonomic function testing enable early detection and intervention [13,76].

### 3.6. Anemia as a Consequence of Congestion in Heart Failure

Anemia is a common complication of chronic congestion in heart failure (HF), with prevalence increasing as HF severity progresses [82,83]. The underlying mechanisms involve hemodilution, renal dysfunction, hepatic impairment, systemic inflammation, and malabsorption, all of which contribute to worsening anemia [84].

Hemodilution due to fluid retention reduces hemoglobin concentration, particularly in advanced HF, affecting up to 50–60% of patients. Hepatic congestion elevates hepcidin levels, restricting iron release and absorption, while inflammatory cytokines suppress erythropoietin (EPO) production, further impairing erythropoiesis [85]. Congestion-induced renal hypoxia exacerbates anemia, particularly in patients with coexisting chronic kidney disease (CKD), which leads to decreased EPO synthesis and increased hepcidin levels [86]. Gastrointestinal congestion and inflammation further hinder iron absorption, compounding iron deficiency and anemia in HF patients [87].

Anemia significantly worsens HF by increasing myocardial hypoxia, left ventricular hypertrophy, and myocardial fibrosis. Additionally, anemia-induced neurohormonal activation (SNS and RAAS) contributes to vasodilation, fluid retention, and renal dysfunction, accelerating HF and CKD progression [87]. In cardiac cachexia, nutritional deficiencies further exacerbate anemia, creating a cycle of worsening HF symptoms [85].

Managing anemia in HF requires a multifaceted approach, including iron supplementation (preferably intravenous for functional iron deficiency), erythropoiesis-stimulating agents in select CKD patients, and optimized diuretic therapy to reduce congestion [87,88].

### 3.7. Musculoskeletal System and Skeletal Muscle Dysfunction

Chronic congestion in heart failure (HF) leads to muscle atrophy, weakness, and reduced endurance due to impaired circulation, which limits oxygen and nutrient supply to muscles, causing mitochondrial dysfunction and oxidative stress [89,90]. Sarcopenia, muscle loss, and strength reduction result from poor perfusion, inflammation, and hormonal imbalances, worsening mobility [91]. Autonomic dysfunction and reduced cardiac output further disrupt stability, increasing fall and fracture risk, especially in older adults [92]. Cardiac cachexia, driven by inflammation, hormonal dysregulation, and poor nutrition, accelerates muscle breakdown, decreases exercise tolerance, and causes fatigue. Fluid retention worsens discomfort, while muscle hypoxia and metabolic dysfunction reduce energy utilization, further impairing function. This cycle of inflammation and physical decline highlights the need for targeted interventions to address musculoskeletal complications in HF patients.

### 3.8. Endocrine System and Metabolic Dysregulation

Heart failure (HF) is associated with significant endocrine disturbances, including insulin resistance, thyroid dysfunction, and adrenal insufficiency. Chronic inflammation and hepatic impairment contribute to insulin resistance, increasing the risk of type 2 diabetes mellitus and worsening cardiovascular complications [93,94].

Thyroid dysfunction, particularly low T3 syndrome, is prevalent in HF due to impaired T4 to T3 conversion, resulting in reduced cardiac output and exercise intolerance, further exacerbating disease progression [93]. Additionally, severe congestion may impair adrenal perfusion, leading to dysregulated cortisol and aldosterone secretion, which contributes to fluid retention, electrolyte imbalances, and hemodynamic instability [95].

Dysregulation of the hypothalamic–pituitary–adrenal (HPA) axis leads to elevated cortisol levels, promoting insulin resistance and metabolic dysfunction, which negatively impact HF prognosis [96]. Furthermore, activation of the renin-angiotensin-aldosterone system (RAAS) increases aldosterone secretion, exacerbating sodium retention and fluid overload, thereby worsening HF symptoms [97].

Sex hormone imbalances, particularly low testosterone levels in men, have been linked to muscle wasting, reduced exercise capacity, and increased morbidity and mortality in HF patients [98,99]. Additionally, HF-related metabolic dysregulation results in reduced insulin sensitivity and impaired glucose tolerance, resembling a starvation state that promotes cachexia and muscle wasting, further accelerating disease progression [96].

### 3.9. Immune System and Chronic Inflammation

Congestion in heart failure (HF) weakens the immune system, increasing vulnerability to infections, sepsis, and delayed wound healing. Persistent venous congestion and tissue hypoxia raise pro-inflammatory cytokines like TNF-α, IL-6, and CRP, leading to endothelial dysfunction, muscle wasting, and cardiac remodeling [18,89,100,101,102,103]. These cytokines are released by immune cells such as macrophages and T lymphocytes in response to congestion and injury, while Angiotensin-converting enzyme inhibitors reduce IL-6 levels and improve HF outcomes [103,104,105]. The renin-angiotensin-aldosterone system (RAAS) worsens inflammation and metabolic disturbances, including insulin resistance, promoting cachexia, worsening H F, and complicating disease management [106,107].

### 3.10. Skin and Peripheral Edema

Congestion and capillary leakage in heart failure (HF) lead to chronic skin changes like venous stasis dermatitis, tissue edema, and ulcers [13,41]. Elevated hydrostatic pressure causes skin discoloration and thickening and increases infection risk, while impaired microvascular perfusion hinders wound healing, making patients prone to pressure ulcers [72,108]. Peripheral edema, common in HF, swells the lower extremities, weakening skin integrity and increasing the risk of cellulitis and other dermatological issues [17,109]. The skin may also show pallor or cyanosis from poor perfusion and become cool and clammy as the body compensates for reduced cardiac output [110].

### 3.11. Hepatosplenic and Lymphatic System Dysfunction

Chronic venous congestion affects both the spleen and lymphatic system, leading to splenomegaly and hematologic abnormalities. Spleen dysfunction disrupts platelet sequestration, increasing the risk of bleeding disorders and pancytopenia [111]. Additionally, congestion impairs lymphatic drainage, contributing to fluid retention and exacerbating peripheral edema [112]. The accumulation of stagnant lymphatic fluid promotes immune cell and cytokine buildup in interstitial spaces, intensifying the inflammatory response [112].

Lymphatic congestion further disrupts interstitial fluid drainage, leading to widespread edema, ascites, and persistent volume overload. The complex interaction between lymphatic dysfunction, venous congestion, and inflammation exacerbates systemic congestion, accelerating HF progression [113].

## 4. Therapeutic Strategies in Congestive Heart Failure

The multifaceted nature of congestion in HF underscores its critical role in disease progression. It is not solely the heart that suffers but rather the entire body, as the effects of congestion propagate across organ systems. Acknowledging and understanding the systemic repercussions of congestion is essential to developing more effective therapeutic strategies and improving patient outcomes in heart failure management [2,15,89].

The primary focus of heart failure therapy, whether for heart failure with reduced ejection fraction (HFrEF) or heart failure with preserved ejection fraction (HFpEF), is decongestion, as both conditions present with similar clinical profiles of congestion during acute heart failure (AHF) decompensation. The strong correlation between early administration of intravenous (IV) loop diuretics and reduced in-hospital mortality supports their role as first-line therapy in AHF (Class I, Level of Evidence B) [15,114]. Early initiation of loop diuretics significantly improves dyspnea within six hours, primarily through renal natriuresis and diuresis. However, more than half of HF patients show little or no weight gain before admission, indicating fluid redistribution rather than absolute volume overload. In such cases, escalating diuretic doses may be counterproductive, as it can lead to excessive plasma volume reduction, decreased renal blood flow, heightened neurohormonal activation, and worsening renal function. Instead, the primary goal in this population should be to enhance venous capacitance and reduce cardiac filling pressures [114]. The addition of vasodilators to low-dose diuretics can help achieve this by reducing preload and inducing arterial vasodilation [15,115].

For hemodynamically stable patients, early initiation of neurohormonal blockade, including angiotensin receptor-neprilysin inhibitors (ARNIs), sodium-glucose cotransporter-2 inhibitors (SGLT2i), mineralocorticoid receptor antagonists (MRAs), and beta-blockers, may further lower cardiac filling pressures, improve venous capacitance, and aid in fluid redistribution [114].

The 2021 European Society of Cardiology (ESC) guidelines for HFrEF emphasize four key pharmacological therapies, often referred to as the “four pillars” of heart failure treatment: angiotensin receptor-neprilysin inhibitors (ARNIs), sodium-glucose cotransporter-2 inhibitors (SGLT2is), beta-blockers, and mineralocorticoid receptor antagonists (MRAs) [116].

The PARADIGM-HF trial was a game-changer, demonstrating that sacubitril/valsartan significantly reduced cardiovascular mortality and heart failure hospitalizations compared to enalapril. As a result, ARNIs became the preferred treatment over ACE inhibitors in eligible patients. These agents combine neprilysin inhibition with angiotensin II receptor blockade, enhancing natriuretic peptides while simultaneously suppressing the renin-angiotensin-aldosterone system (RAAS) [117].

SGLT2 inhibitors, originally developed for diabetes management, have shown remarkable benefits in heart failure therapy. The DAPA-HF, EMPULSE, EMPEROR-Reduced, EMPA-REG OUTCOME trials, and many others provided groundbreaking evidence that dapagliflozin and empagliflozin significantly lower the risk of worsening heart failure and mortality, independent of diabetes status. These findings broadened the therapeutic scope of SGLT2 inhibitors, positioning them as an integral component of heart failure treatment [15,117,118,119,120,121].

Beta-blockers such as bisoprolol, carvedilol, and metoprolol succinate are essential in heart failure management, as they reduce myocardial oxygen demand, control heart rate, and improve survival rates. The European Society of Cardiology recommends their use as a key strategy in improving long-term outcomes in HFrEF [116].

MRAs, including spironolactone and eplerenone, antagonize aldosterone, reducing sodium retention, myocardial fibrosis, and endothelial dysfunction. The EMPHASIS-HF trial confirmed their critical role in improving survival, demonstrating that eplerenone significantly reduced mortality and hospitalizations in mildly symptomatic HFrEF patients [114,116].

## 5. Conclusions

Congestion in heart failure (HF) extends far beyond a simple hemodynamic disturbance, representing a complex, multisystem pathology that impacts nearly every organ system. The pathophysiological interactions between renal dysfunction, hepatic congestion, intestinal permeability, immune dysregulation, and musculoskeletal deterioration highlight the extensive and deleterious effects of congestion in HF. These systemic consequences contribute to a progressive worsening of the disease, increasing morbidity, exacerbating functional impairment, and significantly reducing patient quality of life [25,41].

Renal congestion in HF diminishes renal perfusion and impairs sodium and water handling, further exacerbating fluid retention. Hepatic congestion contributes to a cascade of metabolic dysfunction, including reduced detoxification and compromised protein synthesis. Impaired intestinal integrity, coupled with congestion-induced ischemia, promotes inflammation and nutrient malabsorption, further undermining nutritional status and systemic homeostasis. Concurrently, musculoskeletal atrophy, driven by prolonged fluid retention and inflammation, contributes to cachexia, muscle wasting, and frailty. Collectively, these organ-specific disruptions perpetuate the cycle of congestion, exacerbating both the cardiac and systemic manifestations of heart failure [23,54,55].

## Figures and Tables

**Table 1:** Comprehensive heart failure classification.

Classification System	Criteria and Categories	Clinical Implications
Universal Classification	Stage A: **At risk (no structural disease or symptoms)** **Stage B**: Pre-HF (structural disease, abnormal function, elevated biomarkers, no symptoms) **Stage C:** Symptomatic HF (structural/functional abnormality with past or current symptoms) **Stage D**: Advanced HF (persistent symptoms despite optimal treatment, requiring specialized care)	Identifies patients at risk and guides early intervention strategies to prevent progression.
ACC/AHA Stages	**Stage A:** At risk for HF **Stage B**: Pre-HF (structural disease, no symptoms) **Stage C:** Symptomatic HF **Stage D:** Advanced HF (persistent symptoms despite optimal therapy)	Provides a framework for staging HF, aiding in risk stratification and treatment escalation.
NYHA Functional Classification	**Class I:** No limitation on physical activity **Class II:** Mild symptoms with ordinary activity **Class III:** Marked limitation in daily activities **Class IV**: Symptoms present at rest and worsened by any activity	Assesses functional impairment, guiding therapy intensity, and predicting prognosis.

**Table 2:** Summary of renal congestion effects on long term.

Effect	Description
**Acute Kidney Injury (AKI)**	Chronic congestion reduces renal perfusion, making the renal medulla hypoxic and predisposing it to acute tubular necrosis (ATN).
**Fibrosis and Chronic Kidney Disease (CKD)**	Persistent renal venous hypertension promotes inflammation, oxidative stress, and tubulointerstitial fibrosis, progressively leading to structural nephron loss and worsening renal function.
**Altered Drug Metabolism and Toxicity**	Decreased renal clearance in congested kidneys impairs the excretion of medications such as loop diuretics, ACE inhibitors/ARBs, and Digoxin.
**Electrolyte and Acid-Base Imbalances**	Hyperkalemia due to impaired potassium excretion, exacerbated by RAAS inhibitors;
	Hyponatremia
	Metabolic acidosis
**Systemic Inflammation and Endothelial Dysfunction**	Release of pro-inflammatory cytokines (TNF-α, IL-6), contributing to vascular endothelial dysfunction.
	Accumulation of uremic toxins promotes vascular calcification, oxidative stress, and worsening myocardial remodeling.
**Anemia and Erythropoietin Deficiency**	Impaired erythropoietin (EPO) production in congested kidneys leads to anemia of chronic disease, exacerbating fatigue, hypoxia, and cardiac strain.

## Data Availability

Not applicable.

## Abbreviations

The following abbreviations are used in this manuscript:

DOAJ	Directory of open access journals
HF	Heart failure
